# Target-controlled dialysis for antibiotics (TCD-ABx)

**DOI:** 10.1186/s40635-024-00696-7

**Published:** 2024-11-26

**Authors:** Alexander Dejaco, Christoph Dorn, Constantin Lier, Daniel Fleischmann, Alexander Kratzer, Katharina Habler, Michael Paal, Michael Gruber, Johanna Rosenberger, Martin G. Kees

**Affiliations:** 1 Department of Anaesthesiology, University Hospital Regensburg, Franz-Josef-Strauß-Allee 11, 93053 Regensburg, Germany; 2 Institute of Pharmacy, University of Regensburg, Universitätsstraße 31, 93053 Regensburg, Germany; 3 Hospital Pharmacy, University Hospital Regensburg, Franz-Josef-Strauß-Allee 11, 93053 Regensburg, Germany; 4 Institute of Laboratory Medicine, LMU University Hospital, LMU Munich, Marchioninistr. 15, 81377 Munich, Germany

**Keywords:** Target-controlled dialysis, Renal replacement therapy, Intensive care, Hemodialysis, Antibiotics, Therapeutic drug monitoring

## Abstract

**Background:**

Effective antimicrobial therapy is an essential part of intensive care medicine and renal replacement therapy is an important and common intervention which significantly affects the pharmacokinetics of many antimicrobials. This is especially critical for substances with a narrow therapeutic range, creating a dilemma of weighing the risk of toxicity from increased drug exposure against risk of ineffective treatment and promotion of antimicrobial resistance. To address this problem, we investigate a *target-controlled dialysis* by in vitro experiments — a novel technique in which drug is spiked into the dialysis solution to make use of the physicochemical properties of renal replacement therapy for solute transport, with the goal to reduce the risk of inadequate drug exposure.

**Methods:**

Five antibiotics (ceftazidime, meropenem, piperacillin/tazobactam, vancomycin, flucloxacillin) were dialyzed in an in vitro model of continuous veno-venous hemodialysis using 1 L of bovine serum albumin solution as simulated patient plasma compartment. This was done with and without antibiotics in target concentrations added to the dialysis solution, mimicking three clinically relevant scenarios: (i) target-controlled dialysis in a subject with sub-therapeutic drug levels, (ii) target-controlled dialysis in a subject with supra-therapeutic drug levels, and (iii) traditional dialysis of drugs starting at the target concentration. Drug levels were quantified by high-performance liquid chromatography. Additionally, the stability over 24 h of all antibiotics in two typical dialysis solutions was assessed.

**Results:**

Our data shows that with target-controlled dialysis, antibiotic concentrations will change in the desired direction towards the target concentration, depending on the patients’ unbound drug levels in relation to the concentration in the dialysis solution. The desired target concentrations can be induced and maintained, regardless of the initial concentration. Furthermore, the stability tests revealed only a minor and clinically irrelevant loss in drug concentration (all < 10.2%) after 12 h.

**Conclusions:**

We outlined the mechanistic plausibility and provided experimental evidence of the feasibility of the target-controlled dialysis concept, which could help to maintain therapeutic concentrations of many time-dependent antibiotics in critically ill patients under renal replacement therapy. The required stability in dialysis solutions was shown for a set of important antibiotics. The next step will be the prudent application of this concept to patients in clinical trials.

**Supplementary Information:**

The online version contains supplementary material available at 10.1186/s40635-024-00696-7.

## Background

Critically ill patients with severe infections require effective antimicrobial therapy. Inadequate antimicrobial exposure can lead to toxicity, treatment failure, and emergence of antimicrobial resistance. Therefore, maintaining effective antibiotic levels is a constant challenge, particularly in the intensive care unit. Some substances, like aminoglycosides and daptomycin, work best with fluctuating (i.e., high peak and low through) levels, whereas beta-lactam antibiotics need sustained concentrations due to their time-dependent bactericidal activity. With a slightly different rationale, the same is true for vancomycin.

Our understanding of the pharmacokinetics (PK) and pharmacodynamics (PD) of antibiotics in the critical care setting has greatly improved over the past decades [2, 3, 7, 18], and much translational research has been focused on therapeutic drug monitoring (TDM), PK/PD modeling and precision antibiotic therapy [1, 6, 9, 10, 17].

Renal replacement therapy (RRT), particularly continuous veno-venous hemodialysis (CVVHD), is commonly used in intensive care medicine and significantly affects PK in critically ill patients [12]. Drug elimination by CVVHD depends on physicochemical properties and specific parameters of therapy.

Although predicting the effects of RRT on antibiotic plasma concentrations is possible [5] and numerous dosing recommendations exist [11], in practice, achieving target drug concentrations is challenging due to numerous sources of variability, such as filter types, variable flow rates, and unpredictable down-times. This issue is especially critical for drugs with a narrow therapeutic range, like vancomycin.

To address this problem, we propose a novel modification of traditional hemodialysis, which we call target-controlled dialysis (TCD), with the main objective of keeping antibiotic levels within the optimal range throughout RRT. This study demonstrates the concept and identifies prerequisites of TCD through in vitro experiments under controlled conditions.

## Methods

An in vitro study was conducted to investigate the effects of renal replacement therapy with and without target-controlled dialysis, using the following antibiotics: ceftazidime (CAZ), meropenem (MEM), piperacillin (PIP)/tazobactam (TAZ) (during RRT only PIP levels were quantified), vancomycin (VAN), and flucloxacillin (FXN). Furthermore, the stability of the antibiotics was tested in two common dialysis solutions. No human subjects were involved in this study, therefore ethical approval and trial registration were not required.

### Experimental setup

The experiments were carried out on a MultiFiltratePRO^®^ hemodialysis unit (the device and all disposables are manufactured by Fresenius Medical Care GmbH, Bad Homburg, Germany) in CVVHD mode. It was set up with a HDF 1000 Kit, a Polysulfone^®^ Ultraflux^®^ AV 1000S hemofilter (surface 1.8 ${\text{m}}^2$, allows passage of molecules with a molecular weight of up to approximately 30 kDa), and multiBic^®^ dialysis solution (Na^+^ 140 mmol/L, K^+^ 2 mmol/L, Ca^2+^ 1.5 mmol/L, Mg^2+^ 0.5 mmol/L, Cl^−^ 111 mmol/L, $\text {HCO}_3^-$ 35 mmol/L, glucose 1.0 g/L, osmolality 296 mosmol/L), which come with standard Luer–Lock injection ports.

A simulated patient reservoir (SPR) was prepared using 10% phosphate buffer solution and 45 g/L bovine serum albumin (BSA). The SPR was stored in an Erlenmeyer flask and initially titrated to pH 7.4 with addition of 1 M HCl. Temperature was kept at 37 $^\circ$C, the solution was continuously mixed by a magnetic stirrer, and both pH and temperature were monitored throughout the experiment. The opening of the SPR flask was sealed with foil to prevent evaporation.

**Fig. 1:**
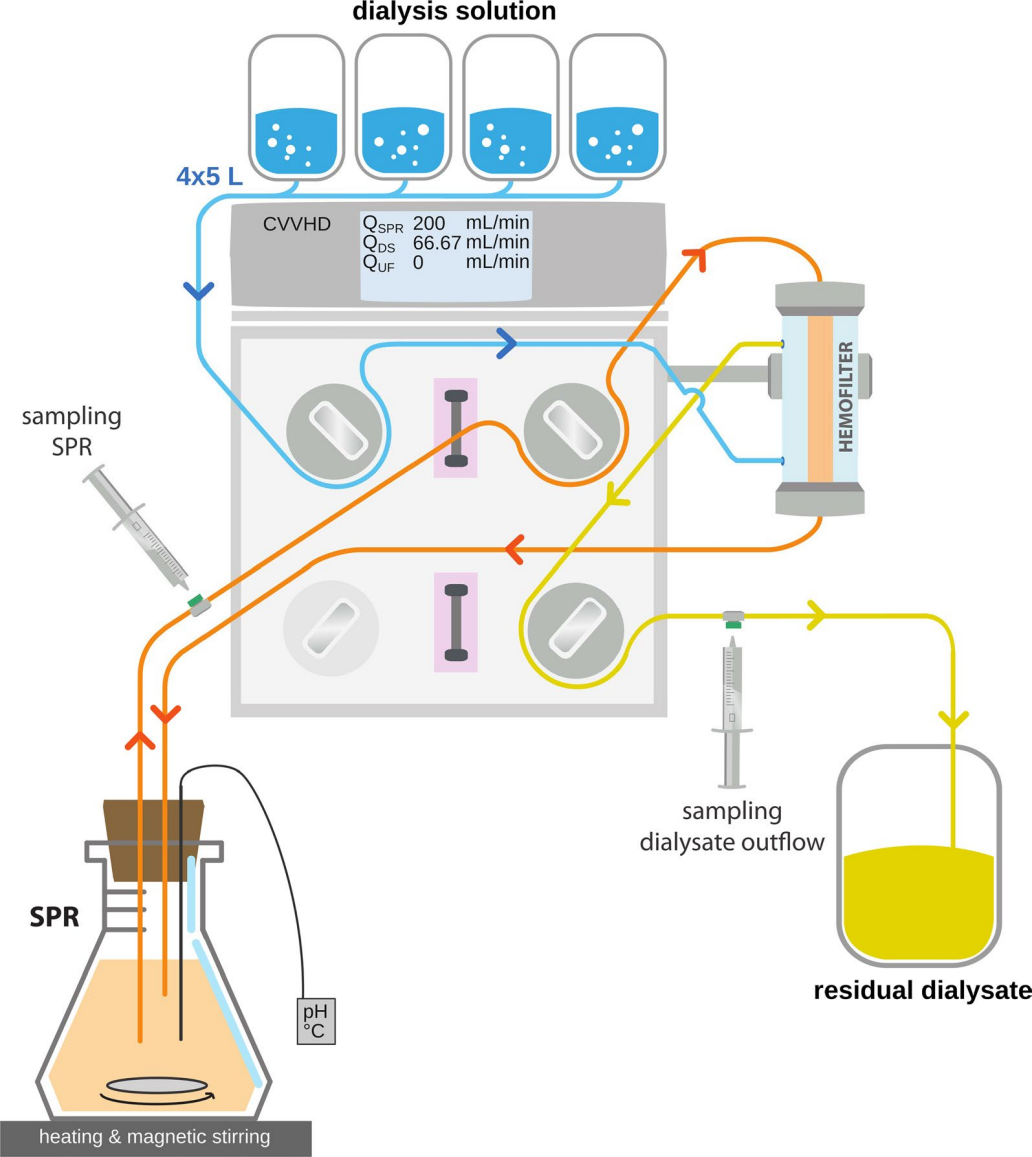
Representation of the experimental setup with a MultiFiltratePRO^®^ hemodialysis unit in continuous veno-venous mode (CVVHD), setup with a hemofilter in counterflow of 1 L simulated patient reservoir (SPR) against dialysis solution (DS) from four parallel 5 L multiBic^®^ dialysis solution bags. SPR flow (“blood flow”) $Q_\text{SPR}$ was set at 200 mL/min, dialysate flow $Q_\text{DS}$ at 66.67 mL/min, and net ultrafiltration $Q_\text{UF}$ at 0 mL/min

The SPR was connected to the MultiFiltratePRO^®^ setup through polyvinyl chloride extension lines attached to the HDF 1000 kit’s tubing, leading to the hemofilter and then back into the SPR. Four standard 5 L bags of multiBic^®^ dialysis solution were connected to the HDF kit tubing, leading to the MultiFiltratePRO^®^ assembly and then to the hemofilter in counterflow direction against the simulated patient fluid (Fig. 1). The outflow line of the hemofilter (i.e., residual dialysate) was connected to standard Fresenius Medical Care dialysate collection bags.

**Fig. 2:**
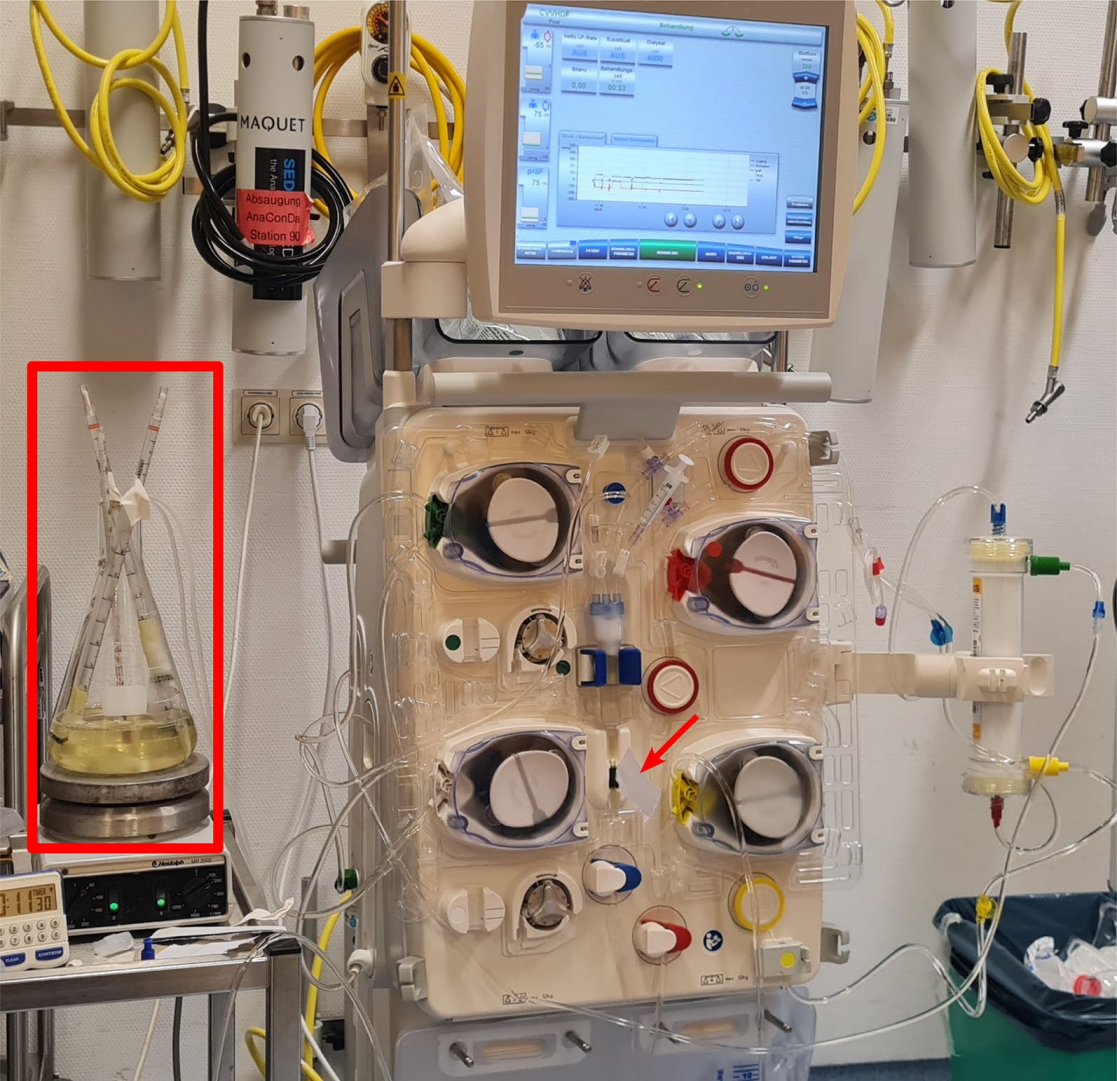
Experimental setup with a MultiFiltratePRO^®^ hemodialysis unit in continuous veno-venous hemodialysis mode, primed and with both in- and outlet tubing attached to a simulated patient reservoir stored in an Erlenmeyer flask (see red rectangle) with continuous mixing by a magnetic stirrer and a heating unit. An obstacle was the machine’s blood detection sensor blocking the initiation of circulation, which was overcome by using a customized piece of cellulose-based fabric (see red arrow)

The circuit was primed using SPR instead of DS or saline solution to prevent subsequent dilution of the SPR when the circulation is activated. Roughly 10 mL of DS was required to fill the device’s drip chamber during the unit’s mandatory initialization procedure. For priming of the circuit, including the hemofilter, approximately 600 mL of SPR fluid were required, which left roughly 400 mL of SPR remaining in the SPR’s container. The primed setup is shown in Fig. 2.

### Experimental protocol

The dialysis experiment was carried out in three phases in order to reflect different scenarios, with simultaneous investigation of all five antibiotics.

- Phase 1: Fresh SPR without antibiotics is dialyzed against spiked DS (i.e., containing antibiotic in TCD target concentrations). This simulates a subject with subtherapeutic antibiotic levels while being initiated on TCD. Subtherapeutic concentrations in the SPR will be increased (“up-dialyzed”) towards the target concentration.
- Phase 2: The SPR is injected with a high dose of antibiotics and dialyzed against spiked DS (again containing antibiotic in TCD target concentrations). This simulates a subject with supratherapeutic antibiotic levels while being on TCD. Supratherapeutic concentrations will be eliminated towards but not below the target concentrations.
- Phase 3: The dialysis solution bags are exchanged with fresh (unspiked) ones. Residual concentrations in the SPR will be eliminated towards zero, simulating traditional dialysis.

**Fig. 3:**
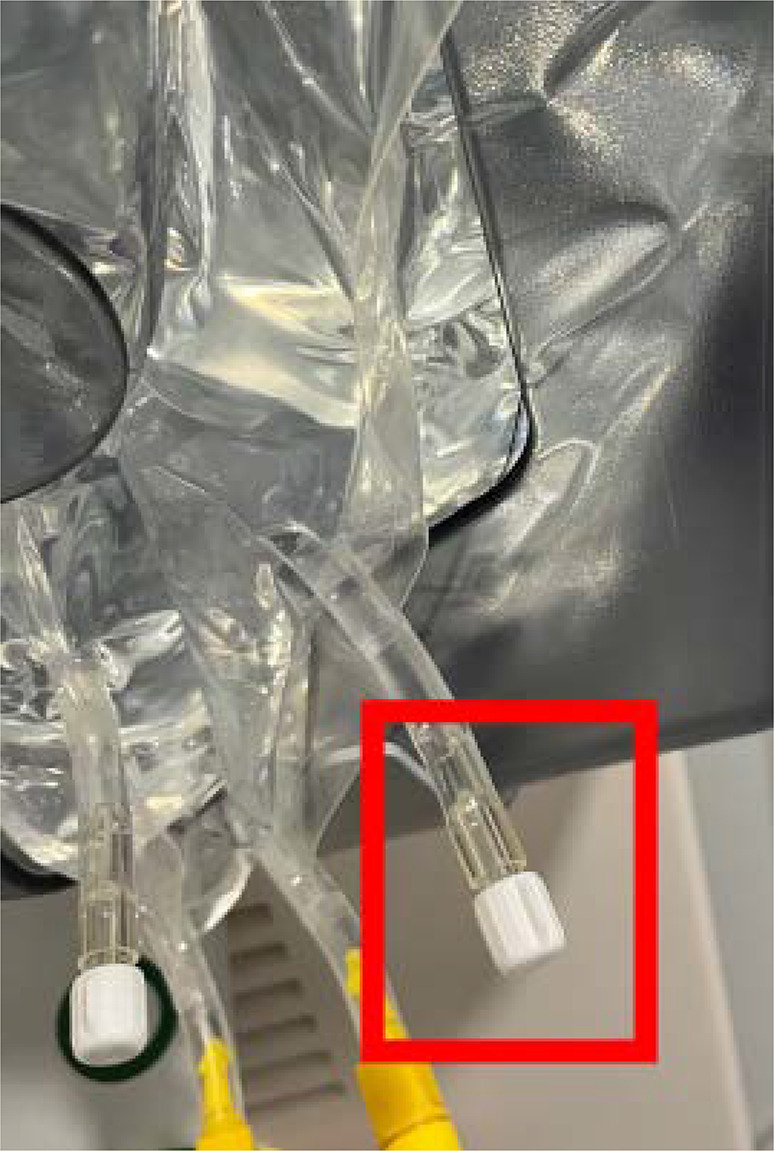
Image of MultiBic^®^ dialysis solution bags, highlighting the standard Luer–Lock access ports, which can be used for safe and simple addition of substances for target-controlled dialysis

To inject antibiotics into the DS, the Luer–Lock ports on the MultiBic^®^ DS bags were used (Fig. 3). The applied antibiotic target concentrations can be found in Table 1. During each phase of the experiment dialysis was carried out in CVVHD mode without interruption for 150 min with a reservoir flow rate ($Q_\text{SPR}$) of 200 mL/min and a dialysis solution flow rate ($Q_\text{DS}$) of 66.7 mL/min, with net ultrafiltration set to zero. In between the three phases, dialysis circulation was briefly paused to change the dialysis solution as well as the residual dialysate bags, and to administer antibiotics, if required according to the protocol. From the SPR and the dialysate outflow, 2 mL samples were taken each at 0, 2.5, 5, 10, 15, 20, 30, 45, 60, 90, 120, and 150 min, during each of the three phases. In addition, initial samples were taken from the DS bags to confirm the TCD target levels. The residual dialysate bags were weighed before the procedure (empty) and at the end of each phase (full), to determine the total amount of spent dialysate.

**Table 1:** Target antibiotic concentrations (mg/L) in 1 L simulated patient reservoir (SPR); concentrations (mg/L) and total amount (mg, in brackets) of spiked drug in four 5 L dialysis solution (DS) bags; for each of three experimental phases

[mg/L] ([mg])	Phase 1		Phase 2		Phase 3	
	SPR 1 L	DS 4 × 5 L	SPR 1 L	DS 4 × 5 L	SPR 1 L	DS 4 × 5 L
Ceftazidime	0	20 (4 × 100)	100	20 (4 × 100)	20	0
Meropenem	0	30 (4 × 150)	90	30 (4 × 150)	30	0
Piperacillin	0	20 (4 × 100)	100	20 (4 × 100)	20	0
Vancomycin	0	15 (4 × 75)	80	15 (4 × 75)	15	0
Flucloxacillin	0	20 (4 × 100)	100	20 (4 × 100)	20	0

### Drug stability

The stability of CAZ, MEM, PIP, TAZ, VAN, and FXN in multiBic^®^ and Ci–Ca^®^ Dialysate K2 (Fresenius Medical Care GmbH, Bad Homburg, Germany) was investigated. The Ci–Ca^®^ solution was not used in the dialysis experiment, but is frequently used in the critical care of patients when regional anticoagulation by citrate is applied. As a control, all substances were also dissolved in 0.9% sodium chloride solution (NaCl), which is recommended as a solvent for all tested drugs. All drugs (concentrations: CAZ and MEM: 40 mg/L, PIP/TAZ: 80/10 mg/L, VAN and FXN: 10 mg/L) were dissolved separately in 5 mL each of both dialysis fluids and NaCl, and incubated for 24 h at room temperature and ambient light. After 0, 3, 6, 12, and 24 h, an aliquot was drawn to check the solutions for any visually detectable precipitates or discolorations and to assess the pH level. Additionally, aliquots for quantification by high-performance liquid chromatography with ultraviolet detection (HPLC-UV) were drawn and immediately frozen at − 80 $^\circ$C to prevent further degradation of the analytes. After thawing and mixing, 10 $\upmu$L were injected into the HPLC system.

### HPLC system

Quantification of drug concentrations was performed by two different laboratories (DF, AK: drug stability testing, CD, CL: dialysis experiments), with slightly different HPLC-UV methods.

To assess drug stability, a Shimadzu Nexera-i LC-2040C 3D system with LabSolution software (Shimadzu Europe, Duisburg, Germany) was used. Detection wavelength was 260 nm (CAZ, MEM, VAN) or 225 nm (PIP, TAZ, FXN). Separation was performed using a Nucleoshell RP18 2.7 $\upmu$m column (i.d. 100 × 3 mm; Macherey-Nagel, Düren, Germany). The mobile phase consisted of 0.1 M sodium phosphate buffer pH 6/acetonitrile 97.5:2.5 (v/v; TAZ), 80:20 (PIP) or 70:30 (FXN), and 0.1 M sodium phosphate buffer pH 2.6/acetonitrile 93:7 (v/v; CAZ, MEM, VAN), respectively. The retention time (flow rate 0.6 mL/min, column temperature 40 $^\circ$C) was 2.6 min (CAZ), 3.4 min (MEM), 4.4 min (VAN), 2.3 min (PIP), 5.0 min (TAZ) and 1.8 min (FXN), respectively.

For the analysis of the drugs in the SPR or DS from the dialysis experiments, a Shimadzu Prominence LC-20 modular system equipped with a photodiode array detector SPD M30A (Shimadzu Europe, Duisburg, Germany) was used. Detection wavelength was 225 nm (PIP, FXN), 240 nm (VAN), 260 nm (CAZ) and 300 nm (MEM), respectively. Separation of CAZ, MEM and VAN was performed using an Avantor ACE C18 3 $\upmu$m column (i.d. 100 × 3 mm, VWR, Darmstadt, Germany) and a mobile phase consisting of 0.1 M sodium phosphate buffer/acetonitrile 92.4:7.6 (v/v), pH 2.9. Separation of PIP and FXN was performed using a Cortecs T3 2.7 $\upmu$m column (i.d. 100 × 3 mm, Waters, Eschborn, Germany) and a mobile phase consisting of 20 mM sodium phosphate buffer/acetonitrile 77:23, (v/v), pH 6.5. The retention time (flow rate 0.4 mL/min, column temperature 40 $^\circ$C) was 4.2 min (CAZ), 5.2 min (MEM), 8.1 min (VAN), 2.3 min (PIP) and 7.6 min (FXN), respectively.

### Sample preparation

Sample preparation for the analysis of total concentrations in the SPR (BSA solution) was performed according to a published protocol [14]. The free concentrations were measured in samples at *t* = 60, 120, 150, 210, and 270 min total experiment duration using a recently published ultrafiltration method prior to HPLC analysis [16]. The unbound fraction was calculated as $f_\text{u} = \frac{C_{\text{free}}}{C_{\text{total}}}$. DS was injected directly into the HPLC system. Injection volume was 1 $\upmu$L for all samples. The lower limit of quantification was conservatively estimated to be $\le$ 0.25 mg/L. Based on spiked quality control samples, intra- and inter-assay imprecision as well as inaccuracy (bias) were < 8%. Regarding the free drug in BSA, accuracy cannot be specified, as the extent of protein binding in a particular sample is not known. The precision was assessed by analysing spiked BSA samples: in these samples the $f_{\text{u}}$ (mean ± SD) was 99.0 ± 0.7% (CAZ), 99.2 ± 1.1% (MEM), 85.0 ± 0.4% (PIP), 79.0 ± 1.1% (VAN) and 16.0 ± 1.0% (FXN), respectively. A comparison of these values with the corresponding unbound fractions in human serum is presented in the electronic supplement, Table A1.

### Data analysis

Data were first recorded in LibreOffice Calc 7.3 and then processed, analyzed, and plotted in Python 3 with JupyterLab 4.0 and matplotlib 3.5.2.

## Results

### Target-controlled dialysis

**Fig. 4:**
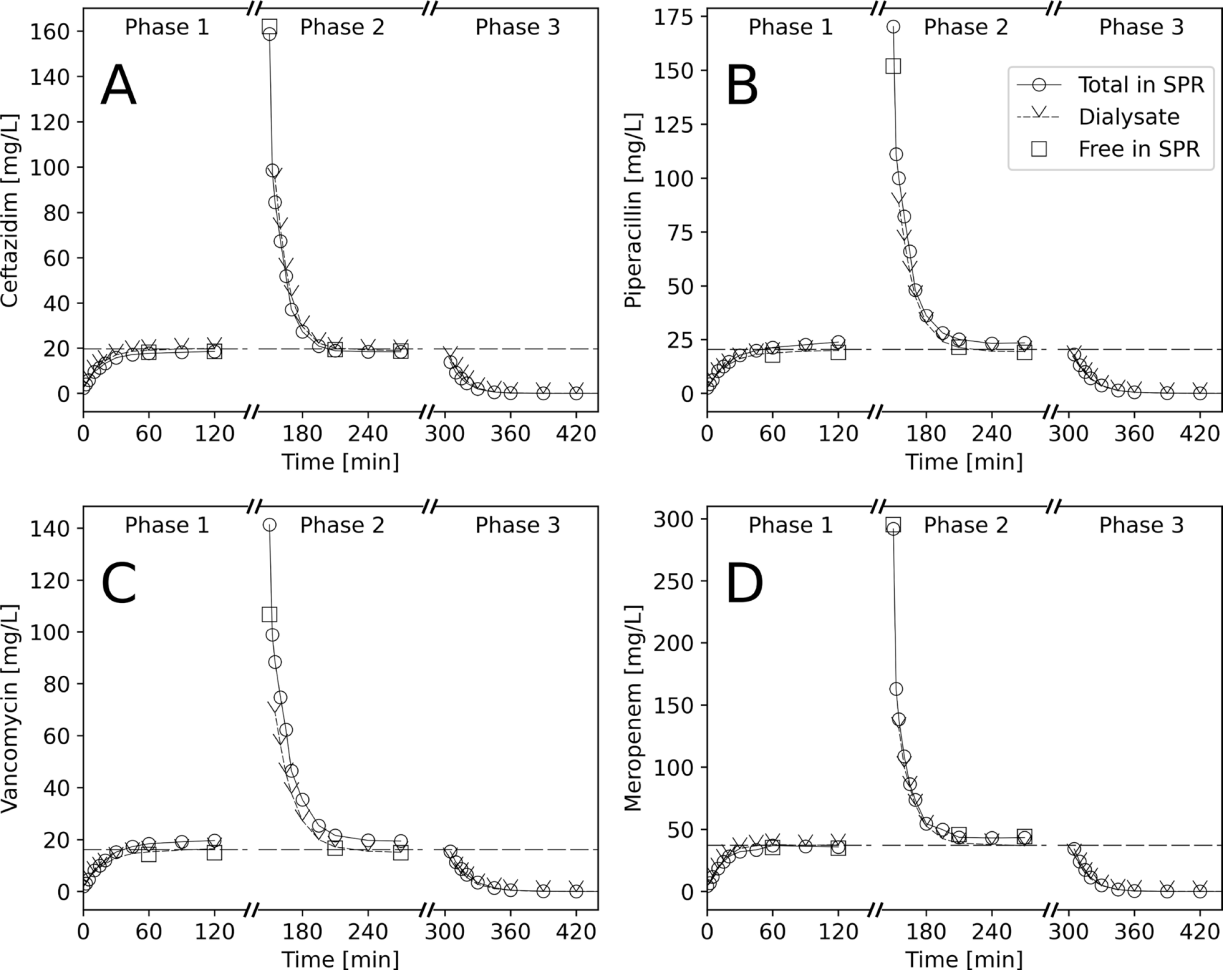
Concentration-time profiles of ceftazidime (**A**), piperacillin (**B**), vancomycin (**C**), and meropenem (**D**) during three sequential phases of an in vitro hemodialysis experiment: phase 1 simulates an antibiotic-naive subject receiving antibiotic through target-controlled dialysis (TCD), phase 2 simulates administration of a high dose of antibiotic with subsequent TCD, and phase 3 shows traditional dialysis. The dashed horizontal line represents the TCD target concentration, i.e., the concentration in the dialysis solution during phases 1 and 2

The effective TCD targets during the experiment (drug levels in the dialysis fluid bags during phases 1 and 2—dashed horizontal lines in Fig. 4) were: 19.6 mg/L for CAZ, 37.3 mg/L for MEM, 20.4 mg/L for PIP, 16.1 mg/L for VAN, and 20.4 mg/L for FXN. The averaged unbound fractions were 102.1% for CAZ, 100.2% for MEM, 83.8% for PIP, 76.6% for VAN, and 12.9% for FXN. Temperature and pH remained stable at 37 $^\circ$C and 7.4, respectively.

**Fig. 5:**
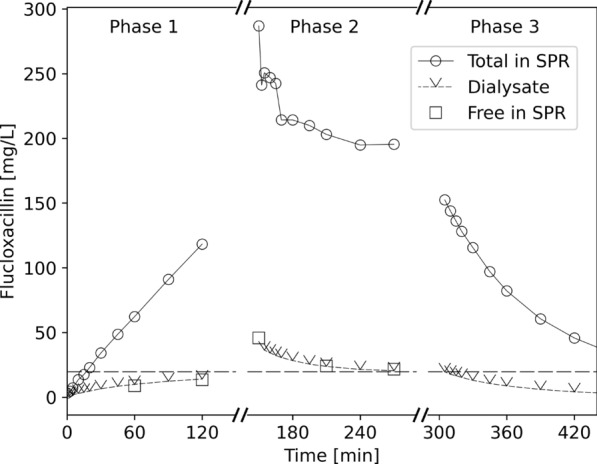
Concentration-time profile of flucloxacillin, an antibiotic highly bound to albumin, during three sequential phases of an in vitro hemodialysis experiment: phase 1 simulates an antibiotic-naive subject receiving antibiotic through target-controlled dialysis (TCD), phase 2 simulates administration of a high dose of antibiotic with subsequent TCD, and phase 3 shows traditional dialysis. The dashed horizontal line represents the TCD target concentration, i.e., the concentration of flucloxacillin in the dialysis solution during phases 1 and 2

Concentration-time curves of CAZ, MEM, PIP, and VAN can be seen in Fig. 4. With all substances, the concentration in the SPR rose or fell quickly towards equilibrium with the target concentration in the DS in phase 1 and 2, respectively, the final concentrations of unbound drugs and those in the dialysate being nearly identical to the target concentrations. In phase 3 (“normal” hemodialysis), antibiotics were rapidly eliminated from the SPR. Figure 5 shows the concentration-time profile of the highly protein-bound FXN. Here, equilibrium was not entirely reached during the experiment, but it is obvious that it is the unbound rather than the total concentration in the SPR which equilibrates with the target concentration in the DS.

Results from additional experiments with slight variations in experimental conditions and results for further substances, as well as a mathematical description of the TCD principle are provided in the electronic supplement (Appendixes A.2 and A.3).

### Drug compatibility and stability

**Fig. 6:**
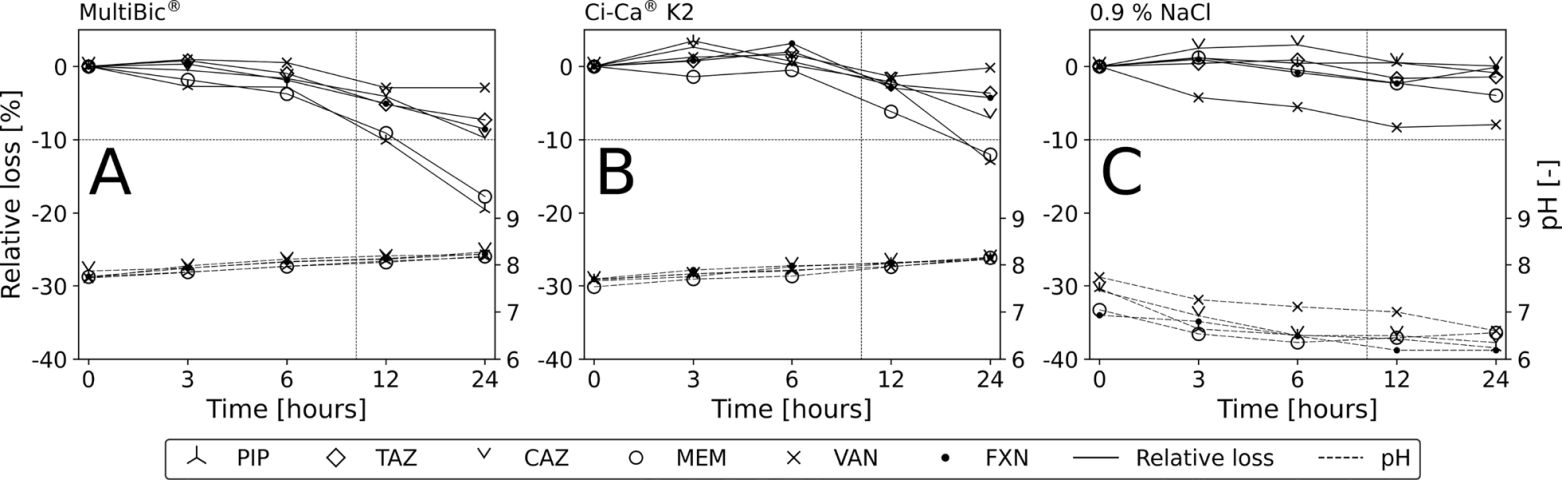
Stability of piperacillin (PIP), tazobactam (TAZ), ceftazidime (CAZ) meropenem (MEM), vancomycin (VAN), and flucloxacillin (FXN) was tested in two typical dialysis solutions: multiBic^®^ (**A**) and Ci–Ca^®^ K2 (**B**), as well as in 0.9% NaCl over 24 h (**C**). Solid lines depict the relative loss (degradation) of drug concentration, and dashed lines pH levels during an incubation period of 24 h. The typical duration of use of dialysis solution bags during continuous veno-venous renal replacement therapy of 10 h is shown by a dotted vertical, and the 10% degradation limit as dotted horizontal line

Visually, no precipitation or discoloration was identified and pH remained stable over the entire incubation period of 24 h for all samples. After 12 h (10 h would comprise the typical duration of four dialysis solution bags of 5 L each with standard settings of 2 L/h dialysate flow rate), the highest observed relative loss was seen for PIP in multiBic^®^ solution with 10.2%. Generally, stability appeared to be highest in NaCl and lowest in multiBic^®^, with Ci–Ca^®^ K2 in between. The relative loss over 24 h is depicted in Fig. 6.

## Discussion

We suggest a simple solution which can help to maintain therapeutic plasma concentrations of certain antibiotics during renal replacement therapy. We outline its mechanistic plausibility and provide first experimental evidence of its feasibility. Modern dialysis devices such as the MultiFiltratePRO^®^ are highly efficient and versatile, but the underlying physicochemical processes of dialysis are essentially quite simple. Plasma and dialysis solution get in contact through a semi-permeable filter, and solutes small enough to pass through that filter will move from the side with the higher concentration towards that with the lower concentration. Solute flow is a function of the difference in concentration and the filter’s characteristics. Although dialysis is traditionally thought of as a method to remove things (excess water, urea, other toxins) from the body, its primary role is to maintain homeostasis. Consequently, dialysis solutions are formulated with approximately physiological concentrations of electrolytes and glucose. For instance, a hyponatremic and hypoglycemic patient treated with dialysis would overall still receive an influx of sodium and glucose through dialysis, as long as levels in the dialysis solution are higher than in the patient’s plasma.

The mechanistic principle of TCD is therefore by no means new — we, however, propose to add concentrations of therapeutic drugs to the dialysis solutions for optimal antibiotic exposure and therapeutic success in critically ill patients. Given the importance of anti-infective therapy, we addressed commonly used antibiotics as the first candidates, but other drug classes might be suitable as well, e.g., antiepileptics. Generally, suitable scenarios to consider for TCD are drugs which undergo neglectable extrarenal elimination, exert their optimal efficacy with an even, non-fluctuating concentration-time profile and are given to patients without relevant residual renal function.

Our in vitro experiment confirms that TCD operates according to the same well-established and straightforward mechanistic principles as traditional dialysis [4]. Specifically, (i) the tested antibiotics move in both directions, and (ii) it is the concentration of unbound drug in plasma which equilibrates with the concentration in the (protein-free) dialysis solution. The latter aspect is also in accordance with fundamental assumptions on the pharmacology of drugs: only the unbound fraction is pharmacologically active, can bind to receptors or be bio-transformed, eliminated, diffuse to other compartments and so on. Whereas for CAZ, MEM, PIP, and VAN the difference is small, it cannot be overseen in the case of FXN. At first glance, this could be problematic, when deciding on reasonable target concentrations for TCD, because physicians mostly think in total concentrations. However, overcoming this habit would be preferable, since pharmacotherapeutic adjustments based on total concentrations (only) do not account for inter-individual variability of plasma protein binding, which is high for some drugs (e.g., FXN) [19].

Before moving forward to clinical trials, drug stability in dialysis solutions must be demonstrated. Our respective experiments demonstrated only minor degradation of $\le$10.2% over 12 h, which is longer than the expected run-time for one set of DS (four bags of 5 L each with a typical dialysate flow rate of 2 L/h, i.e., 10 h). We would not expect this degradation to have any clinical impact. The internationally accepted threshold for drug stability testing lies at 90% drug content [13].

Finally, suitable target concentrations for the use in patients must be selected. Fundamental and widely accepted PK/PD principles can be used as guidance, as has been done in studies on continuous infusion with or without TDM of betalactams [10]. Concentrations of about the fivefold of the susceptibility breakpoint or epidemiological cut off value as defined by the European Committee on Antimicrobial Susceptibility Testing [8] of relevant target pathogens (*Pseudomonas aeruginosa* for CAZ, MEM and PIP, Staphylococcus aureus for FXN) would yield 40 mg/L for CAZ and MEM, 80 mg/L for PIP and 10 mg/L for FXN. For VAN, the suggested target of 20–25 mg/L during continuous infusion would translate into 15–20 mg/L of unbound concentration, assuming a protein binding rate of 25–30%. It can be safely assumed that somewhat lower or higher target concentrations would perform equally well in TCD-ABx.

Although our experiment focused on simulating CVVHD, the underlying principle could, in theory, extend to other forms of dialysis, such as sustained low-efficiency dialysis, and even hemofiltration, where ultrafiltrate would be substituted with dialysis/substitution solution containing the drug in the target concentration. Clinically, TCD has the potential to replace conventional antibiotic dosing in the long run (as suggested in phase 1 of Figs. 4 and 5). The optimization of antibiotic dosing strategies during RRT in critically ill patients, particularly in the ICU, remains a subject of ongoing debate [12, 15]. TCD could offer significant simplification of antibiotic therapy during RRT. Thus, the primary advantage of TCD, in our view, lies in enhancing the safety and efficacy of therapy in patients undergoing dialysis. To validate this proof of concept, clinical trials will be essential to assess its applicability in the complex environment of critically ill patients. The next step, however, will be clinical case series of TCD under rigorous monitoring, before the initiation of larger trials. From a practical perspective, integrating TCD into clinical practice should be straightforward. The dialysis solution bags we used feature a standard Luer–Lock port (Fig. 3), which ensures that drug administration is both safe and simple. Of course, for successful implementation of TCD, the logistics of spiking and mixing of dialysis fluid must be carefully managed and well established to ensure correct and homogeneous drug concentrations within the solution. With typical CVVHD settings, the need for bag changes is minimal, generally requiring no more than one or two changes per ICU shift.

Our study has several limitations, beyond the general ones intrinsic to in vitro experiments. We used only a small volume of 1 L to simulate the patient. We recognize that this is at least an order of magnitude lower than the $V_d$ seen in critically ill patients and cannot mimic drug distribution between different compartments (e.g., blood, interstitial, or target organs). The simplified model was chosen deliberately to limit costs, the duration of a single experiment, and to focus on demonstrating the core mechanism of TCD. Importantly, the volume of distribution affects only the time required to reach equilibrium, not the target equilibration concentration itself. The effect of $V_d$ on equilibration kinetics is easily predictable from basic pharmacokinetic formulae ($t_{1/2} = \text{ln} 2 \times V_d/\text{Cl}$). A larger $V_d$ will extend the time to equilibrium, but would not alter the eventual equilibrium concentration.

The dialysis parameters and flow rates which determine clearance were similar to those used in clinical routine, resulting in a very short half-life of drug kinetics and rapid equilibration.

We used a cell-free solution of bovine serum albumin in phosphate buffered saline for reasons of costs and simplicity. However, we have compared the unbound fractions of the investigated drugs in the BSA solution against human serum (electronic supplement, Table A1).

While we performed no replicate TCD experiments with identical conditions, we varied the drugs and concentrations and performed several preliminary experiments with slightly varying conditions and RRT settings as well as voriconazole as an additional substance with substantial protein binding (electronic supplement, Figures A1–A5) in preparation to the presented study, to create as reliable conditions as possible in the final experiment.

A key advantage of TCD is that its mechanism operates independently of patient centered variables like tissue binding and metabolic processes. It is uniquely suited to address, e.g. variability in protein binding, as it directly targets the free, pharmacologically active concentration. Unlike traditional TDM, which relies mostly on total concentrations and can therefore be sensitive to changes in protein binding in the critically ill.

## Conclusion

We demonstrated in a very simple in vitro model the feasibility and fundamental requirements of target-controlled dialysis, which could facilitate maintaining therapeutic concentrations of many time-dependent antibiotics in critically ill patients under renal replacement therapy. The necessary stability for a set of important betalactam antibiotics and vancomycin in dialysis solutions was shown. The next step will be prudent and stepwise (e.g. short-term) application in clinical trials in patients.

## Supplementary Information

Supplementary Material 1.

## Data Availability

The data sets used and/or analyzed during the current study are available from the corresponding author on reasonable request.

## Abbreviations

TCD: Target-controlled dialysis
PK: Pharmacokinetics
PD: Pharmacodynamics
TDM: Therapeutic drug monitoring
RRT: Renal replacement therapy
CVVHD: Continuous veno-venous hemodialysis
CAZ: Ceftazidime
MEM: Meropenem
PIP: Piperacillin
TAZ: Tazobactam
VAN: Vancomycin
FXN: Flucloxacillin
DS: Dialysis solution
SPR: Simulated patient reservoir
BSA: Bovine serum albumin
HPLC-UV: High-performance liquid chromatography with ultraviolet detection
SD: Standard deviation
$$f_\text{u}$$: Unbound fraction
$Q_{\text{SPR}}$: Simulated patient reservoir flow rate
$Q_{\text{DS}}$: Dialysate flow rate
$Q_{\text{UF}}$: Ultrafiltration flow rate
$C_{\text{free}}$: Concentration of free (unbound) substance
$C_{\text{total}}$: Total concentration of substance (free and bound to protein)

## References

[1] Abdulla A, Ewoldt TMJ, Hunfeld NGM et al (2020) The effect of therapeutic drug monitoring of beta-lactam and fluoroquinolones on clinical outcome in critically ill patients: the DOLPHIN trial protocol of a multi-centre randomised controlled trial. BMC Infect Dis 20:57. 10.1186/s12879-020-4781-x 31952493 10.1186/s12879-020-4781-x PMC6969462

[2] Asín-Prieto E, Rodríguez-Gascón A, Isla A (2015) Applications of the pharmacokinetic/pharmacodynamic (PK/PD) analysis of antimicrobial agents. J Infect Chemother 21(5):319–329. 10.1016/j.jiac.2015.02.001 25737147 10.1016/j.jiac.2015.02.001

[3] Berry AV, Kuti JL (2022) Pharmacodynamic thresholds for beta-lactam antibiotics: a story of mouse versus man. Front Pharmacol 13:833189. 10.3389/fphar.2022.833189 35370708 10.3389/fphar.2022.833189 PMC8971958

[4] Böhler J, Donauer J, Keller F (1999) Pharmacokinetic principles during continuous renal replacement therapy: drugs and dosage. Kidney Int 56:S24–S28. 10.1046/j.1523-1755.56.s.72.2.x 10560800

[5] Broeker A, Vossen MG, Thalhammer F et al (2020) An integrated dialysis pharmacometric (IDP) model to evaluate the pharmacokinetics in patients undergoing renal replacement therapy. Pharm Res 37(6):96. 10.1007/s11095-020-02832-w 32409892 10.1007/s11095-020-02832-w PMC7225193

[6] Bulman ZP, Wicha SG, Nielsen EI et al (2022) Research priorities towards precision antibiotic therapy to improve patient care. Lancet Microbe 3(10):e795–e802. 10.1016/S2666-5247(22)00121-5 35777386 10.1016/S2666-5247(22)00121-5

[7] Crass RL, Rodvold KA, Mueller BA et al (2019) Renal dosing of antibiotics: are we jumping the gun? Clin Infect Dis 68(9):1596–1602. 10.1093/cid/ciy790 30219824 10.1093/cid/ciy790

[8] EUCAST (2024) The European Committee on Antimicrobial Susceptibility Testing. Breakpoint tables for interpretation of MICs and zone diameters. Version 14.0. https://www.eucast.org/clinical_breakpoints. Accessed 10 June 2024

[9] Greppmair S, Brinkmann A, Roehr A et al (2023) Towards model-informed precision dosing of piperacillin: multicenter systematic external evaluation of pharmacokinetic models in critically ill adults with a focus on Bayesian forecasting. Intensive Care Med 49(8):966–976. 10.1007/s00134-023-07154-0 37439872 10.1007/s00134-023-07154-0 PMC10425489

[10] Hagel S, Bach F, Brenner T et al (2022) Effect of therapeutic drug monitoring-based dose optimization of piperacillin/tazobactam on sepsis-related organ dysfunction in patients with sepsis: a randomized controlled trial. Intensive Care Med 48(3):311–321. 10.1007/s00134-021-06609-6 35106617 10.1007/s00134-021-06609-6 PMC8866359

[11] Heintz BH, Matzke GR, Dager WE (2009) Antimicrobial dosing concepts and recommendations for critically ill adult patients receiving continuous renal replacement therapy or intermittent hemodialysis. Pharmacotherapy 29(5):562–577. 10.1592/phco.29.5.562 19397464 10.1592/phco.29.5.562

[12] Hoff BM, Maker JH, Dager WE et al (2020) Antibiotic dosing for critically ill adult patients receiving intermittent hemodialysis, prolonged intermittent renal replacement therapy, and continuous renal replacement therapy: an update. Ann Pharmacother 54(1):43–55. 10.1177/1060028019865873 31342772 10.1177/1060028019865873

[13] ICH Expert Working Group (2003) ICH topic Q1A (R2) stability testing of new drug substances and products—scientific guideline. https://www.ema.europa.eu/en/ich-q1a-r2-stability-testing-new-drug-substances-drug-products-scientific-guideline. Accessed 14 June 2024

[14] Kratzer A, Schießer S, Matzneller P et al (2019) Determination of total and free ceftolozane and tazobactam in human plasma and interstitial fluid by HPLC-UV. J Pharm Biomed Anal 163:34–38. 10.1016/j.jpba.2018.09.044 30278324 10.1016/j.jpba.2018.09.044

[15] Lee A, De Waele JJ, Lipman J (2022) Antibiotic dosing in sustained low-efficiency daily dialysis (SLEDD): basic concepts and dosing strategies. J Crit Care 67:104–107. 10.1016/j.jcrc.2021.10.019 34741962 10.1016/j.jcrc.2021.10.019

[16] Lier C, Dejaco A, Kratzer A et al (2024) Free serum concentrations of antibiotics determined by ultrafiltration: extensive evaluation of experimental variables. Bioanalysis. 10.1080/17576180.2024.2365526 39041640 10.1080/17576180.2024.2365526 PMC11389746

[17] Rybak MJ, Le J, Lodise TP et al (2020) Therapeutic monitoring of vancomycin for serious methicillin-resistant *Staphylococcus aureus* infections: a revised consensus guideline and review by the American society of health-system pharmacists, the infectious Diseases Society of America, the Pediatric Infectious Diseases Society, and the Society of Infectious Diseases pharmacists. Am J Health-Syst Pharm 77(11):835–864. 10.1093/ajhp/zxaa036 32191793 10.1093/ajhp/zxaa036

[18] Udy AA, Dulhunty JM, Roberts JA et al (2017) Association between augmented renal clearance and clinical outcomes in patients receiving beta-lactam antibiotic therapy by continuous or intermittent infusion: a nested cohort study of the BLING-II randomised, placebo-controlled, clinical trial. Int J Antimicrob Agents 49(5):624–630. 10.1016/j.ijantimicag.2016.12.022 28286115 10.1016/j.ijantimicag.2016.12.022

[19] Wallenburg E, Brüggemann RJM, Roberts JA et al (2022) A meta-analysis of protein binding of flucloxacillin in healthy volunteers and hospitalized patients. Clin Microbiol Infect 28(3):446.e1-446.e7. 10.1016/j.cmi.2021.06.039 34245903 10.1016/j.cmi.2021.06.039

