# Supplementary material for: Target-controlled dialysis for antibiotics (TCD-ABx)
Source: Intensive Care Med Exp. 2024 Nov 26;12:108. doi: 10.1186/s40635-024-00696-7 (PMC11599528; doi:10.1186/s40635-024-00696-7)
Supplement: Supplementary file 1 — Supplementary Material 1. [file 40635_2024_696_MOESM1_ESM.pdf]

Target-Controlled Dialysis for Antibiotics (TCD-ABx)

Alexander Dejaco[1*], Christoph Dorn[2], Constantin Lier[2], Daniel Fleischmann[3], Alexander Kratzer[3], Katharina Habler[4], Michael Paal[4], Michael Gruber[1], Johanna Rosenberger[1], Martin G. Kees[1]

[1*]Department of Anaesthesiology, University Hospital Regensburg, Franz-Josef-Strauß-Allee 11, Regensburg, 93053, Germany.
[2]Institute of Pharmacy, University of Regensburg, Universitätsstraße 31, Regensburg, 93053, Germany.
[3]Hospital Pharmacy, University Hospital Regensburg, Franz-Josef-Strauß-Allee 11, Regensburg, 93053, Germany.
[4]Institute of Laboratory Medicine, LMU University Hospital, LMU Munich, Marchioninistr. 15, Munich, 91377, Germany.

*Corresponding author(s). E-mail(s): alexander.dejaco@klinik.uni-regensburg.de;
Contributing authors: christoph.dorn@chemie.uni-regensburg.de; constantin.lier@chemie.uni-regensburg.de; daniel.fleischmann@klinik.uni-regensburg.de; alexander.kratzer@klinik.uni-regensburg.de; katharina.habler@med.uni-muenchen.de; michael.paal@med.uni-muenchen.de; michael.gruber@klinik.uni-regensburg.de; johanna.rosenberger@klinik.uni-regensburg.de; martin.kees@klinik.uni-regensburg.de;

Appendix A Supplementary data

A.1 Unbound fractions in bovine serum albumin solution and human serum

Table A1 Unbound fraction (fu) of different drugs from preparatory experiments for in vitro target-controlled dialysis. The fu is shown for the investigated drugs (at concentrations 50 and 100 mg/L) as determined in human serum (left column) or in PBS (pH 7.4) containing 4.5 % bovine serum albumin (BSA; middle column). The fu as provided by the summary of product characteristics (SmPC) is given in the right column.

	fu (experiments)				fu (SmPC)
	Human serum[1]		4.5% BSA		
	mean ± SD [%]	n [-]	mean ± SD [%]	n [-]	[%]
CAZ	98 ± 0.6	6	99 ± 0.7	4	≈ 90
MEM	98 ± 0.4	6	99 ± 1.1	4	≈ 98
PIP	91 ± 0.5	6	85 ± 0.4	6	≈ 70
VAN	74 ± 2.7	6	79 ± 1.1	4	≈ 45-70
FXN	4 ± 0.5	6	16 ± 1.0	6	≈ 4-8
VRC	51 ± 1.8	4	40 ± 0.6	3	≈ 42

[1]Data already published by Lier et al (2024)

A.2 Supplementary data from additional experiments

As expected, the higher volume of distribution of 3 L (experiment 1) compared to 1 L (experiments 2 and 3) together with lower "blood" and dialysate flow rates (experiment 1) resulted in a significantly longer half-life, in line with pharmacokinetic principles (Figures A1-A5).

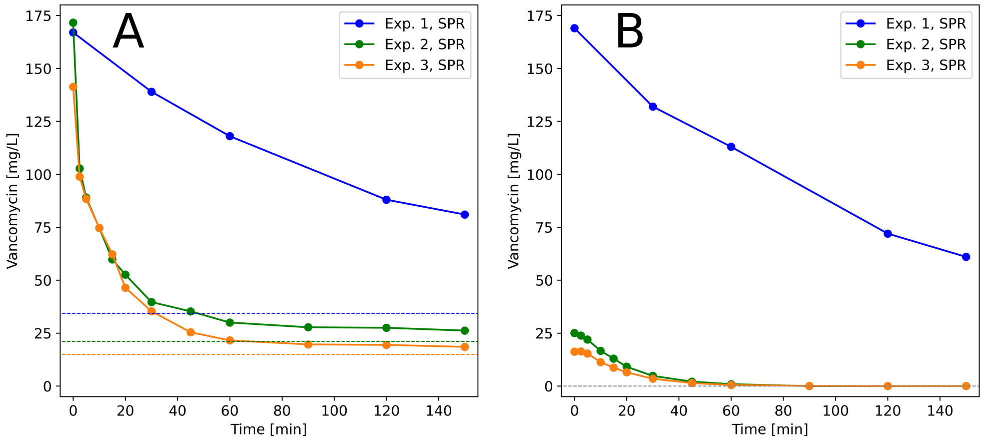

Fig. A1 Vancomycin (VAN) concentrations during three in vitro experiments of target-controlled dialysis (TCD) conducted under slightly different conditions and with two consecutive phases (A and B). Exp. 1: VAN is dialyzed from a 3 L simulated patient reservoir (SPR) against concentrations of 34.4 mg/L (A) and 0 mg/L (B) in the dialysis solution (DS), with a "blood" flow rate (Q_{SPR}) of 100 mL/min and a dialysate flow rate Q_{DS} of 33.4 mL/min; Exp. 2: VAN is dialyzed from a 1 L SPR against concentrations of 21.1 mg/L (A) and 0 mg/L (B) in DS, with a Q_{SPR} of 200 mL/min and a Q_{DS} of 66.7 mL/min; Exp. 3: VAN is dialyzed from a 1 L SPR against concentrations of 15 mg/L (A) and 0 mg/L (B) in DS, with a Q_{SPR} of 200 mL/min and a Q_{DS} of 66.7 mL/min.

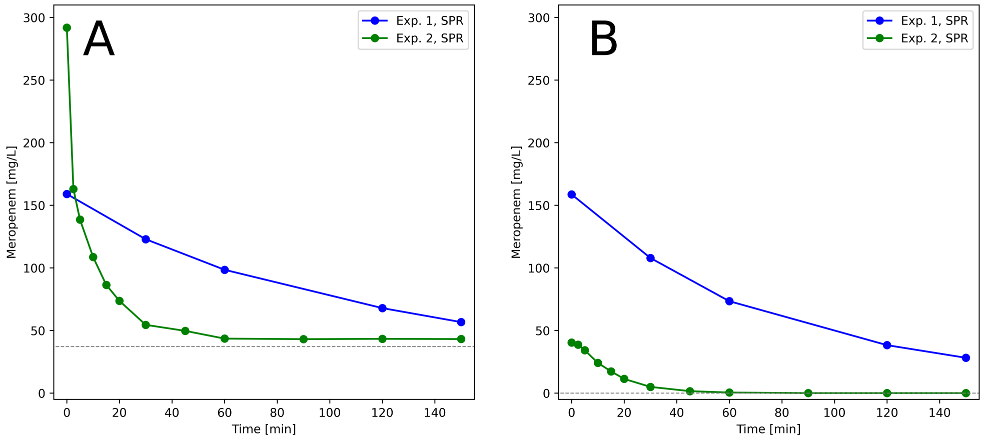

Fig. A2 Meropenem (MEM) concentrations during two in vitro experiments of target-controlled dialysis (TCD) conducted under slightly different conditions and with two consecutive phases (A and B). Exp. 1: MEM is dialyzed from a 3 L simulated patient reservoir (SPR) against concentrations of 37.2 mg/L (A) and 0 mg/L (B) in the dialysis solution (DS), with a "blood" flow rate Q_{SPR} of 100 mL/min and a dialysate flow rate Q_{DS} of 33.4 mL/min; Exp. 2: MEM is dialyzed from a 1 L SPR against concentrations of 37.2 mg/L (A) and 0 mg/L (B) in DS, with a Q_{SPR} of 200 mL/min and a Q_{DS} of 66.7 mL/min.

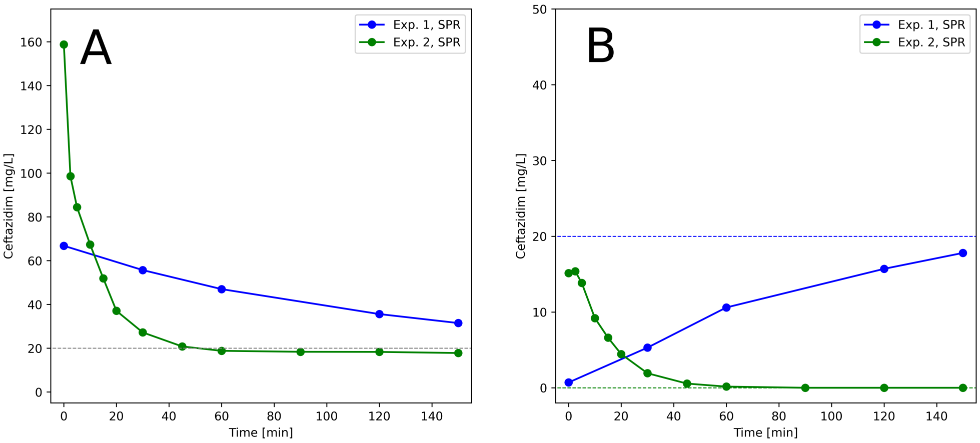

Fig. A3 Concentrations of ceftazidim (CAZ) during two in vitro experiments of target-controlled dialysis (TCD) under slightly different conditions and with two consecutive phases (A and B). Exp. 1: CAZ is dialyzed (A) and introduced (B) from a 3 L simulated patient reservoir (SPR) against concentrations of 20 mg/L in dialysis solution (DS), with a "blood" flow rate Q_{SPR} of 100 mL/min and a dialysate flow rate Q_{DS} of 33.4 mL/min; Exp. 2: CAZ is dialyzed from a 1 L SPR against concentrations of 20 mg/L (A) and 0 mg/L (B) in DS, with a Q_{SPR} of 200 mL/min and a Q_{DS} of 66.7 mL/min.

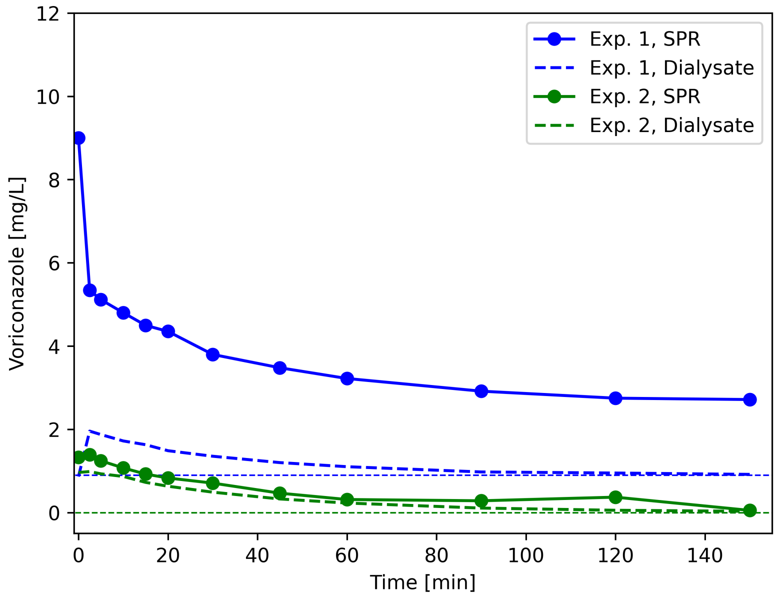

Fig. A4 Concentrations of voriconazole (VRC) during two in vitro experiments of target-controlled dialysis (TCD) under slightly different conditions. VRC is dialyzed from a 1 L simulated patient reservoir (SPR) against a concentration of 0.9 mg/L in the dialysis solution (DS; Exp. 1), and against a concentration of 0 mg/L in DS (Exp. 2) with a "blood" flow rate Q_{SPR} of 200 mL/min and a dialysate flow rate Q_{DS} of 66.7 mL/min. Solid lines represent concentrations of VRC in the SPR (i.e. total concentrations) and dashed lines concentrations of VRC in the dialysate outflow line (i.e. approximately free concentrations).

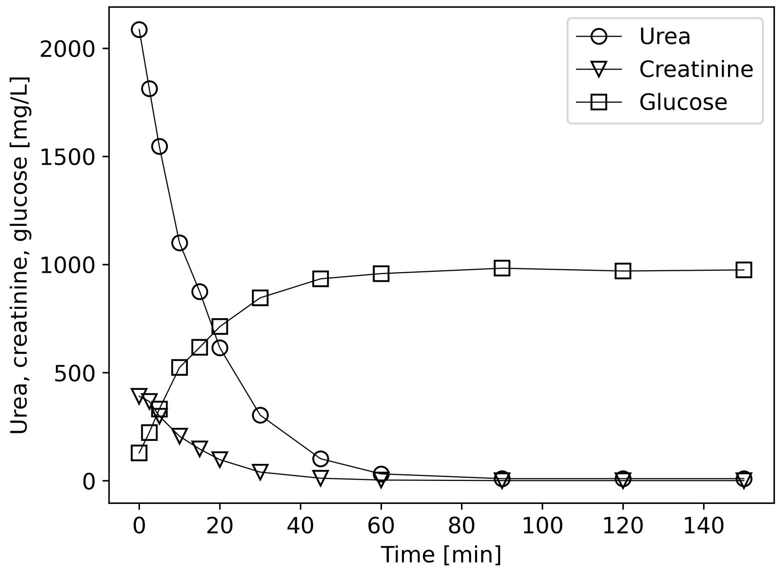

Fig. A5 Concentrations of urea, creatinine and glucose during an in vitro experiment of target-controlled dialysis. Urea was added to the simulated patient reservoir (SPR) in a concentration of 2,500 mg/L and dialyzed against 0 mg/L in the dialysis solution. Creatinine was added to the SPR in a concentration of 400 mg/L and dialyzed against 0 mg/L in the dialysis solution. Glucose was already contained in a concentration of 1,000 mg/L in the original dialysis solution. "Blood" flow rate during dialysis was 200 mL/min and the dialysate flow rate was 33.4 mL/min. Creatinine, urea and glucose were quantified using the Cobas® 8000 standard clinical chemistry analyzer module C702 (Roche Diagnostics, Mannheim, Germany).

A.3 Mathematical description

The principle mechanisms of dialysis follow simple pharmacokinetic (PK) principles Böhler et al (1999). The basic concentration-time profiles of antibiotic concentrations during renal replacement therapy can be approximated by a simple elimination from one compartment. Adapting PK equations for phases 1-3 of the in vitro experiment (Figures 4 and 5) as follows:

- Phase 1 - TCD with retrograde solute transport: $C = C_{\mathrm{TCD}} - C_{\mathrm{TCD}} e^{-kt}$
- Phase 2 - Dialysis against a TCD target: $C = C_0 e^{-kt} + C_{\mathrm{TCD}}$.
- Phase 3 – Normal dialysis: $C = C_0 e^{-kt}$.

with $k = \frac{\mathrm{Cl}}{V_d}$ as the elimination/adsorption rate constant, C_0 as the initial concentration, and C_{TCD} as the TCD target concentration. As an illustration, the elimination/adsorption coefficient k and C_{TCD} was determined by maximum likelihood estimation using an ordinary least squares objective function for our in vitro TCD experiment with piperacillin (Figure 4B) and is shown in Figure A6. This highlights, that TCD follows well known simple pharmacokinetic principles that can be well described.

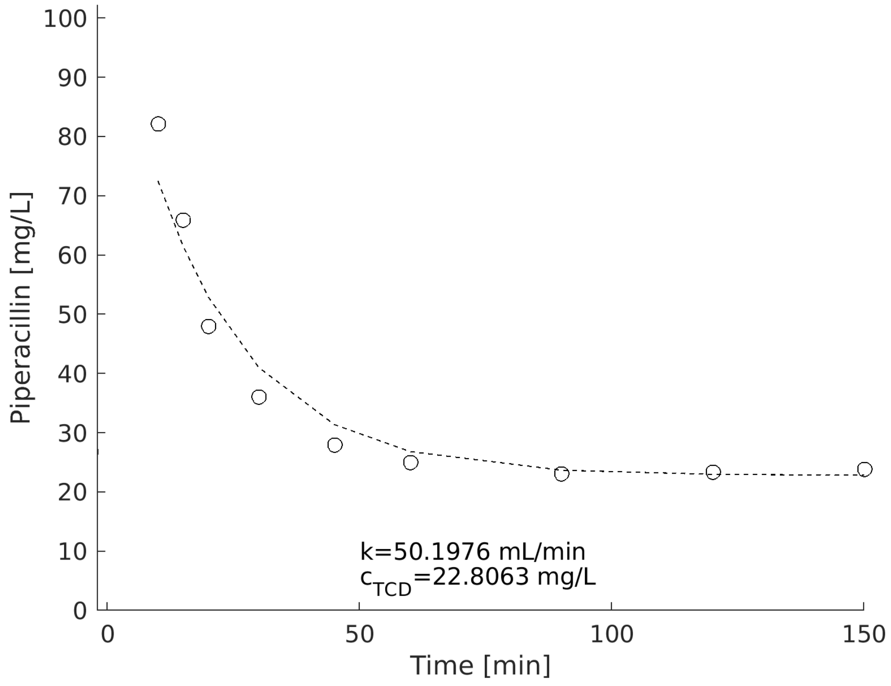

Fig. A6 Estimation of the elimination constant k and the target concentration C_{TCD} by maximum likelihood estimation and the equation $C = C_0 e^{-kt} + C_{\mathrm{TCD}}$ to describe the concentration-time profile of piperacillin during phase 2 of the in vitro TCD experiment (Figure 4).

References

Böhler J, Donauer J, Keller F (1999) Pharmacokinetic principles during continuous renal replacement therapy: Drugs and dosage. Kidney International 56:S24–S28. https://doi.org/10.1046/j.1523-1755.56.s.72.2.x

Lier C, Dejaco A, Kratzer A, et al (2024) Free serum concentrations of antibiotics determined by ultrafiltration: Extensive evaluation of experimental variables. Bioanalysis (In Press). https://doi.org/10.1080/17576180.2024.2365526
